# The Role of *Blastocystis* spp. in the Etiology of Gastrointestinal and Autoimmune Diseases

**DOI:** 10.3390/pathogens14040313

**Published:** 2025-03-25

**Authors:** Oliwia Pawelec-Pęciak, Natalia Łanocha-Arendarczyk, Konrad Grzeszczak, Danuta Kosik-Bogacka

**Affiliations:** 1 Department of Biology, Parasitology and Pharmaceutical Botany, Pomeranian Medical University in Szczecin, Powstanców Wielkopolskich 72, 70-111 Szczecin, Poland; olipaw96@gmail.com (O.P.-P.); danuta.kosik.bogacka@pum.edu.pl (D.K.-B.); 2 Department of Medical Analytics, Pomeranian Medical University in Szczecin, Powstanców Wielkopolskich 72, 70-111 Szczecin, Poland; k.grzeszczak@kidl.org.pl

**Keywords:** *Blastocystis* spp., autoimmune diseases, IBS, CRC, treatment, metronidazole, medicinal plants, probiotics

## Abstract

*Blastocystis* spp. has been linked to gastrointestinal symptoms, yet its pathogenicity remains uncertain. In addition, the roles of virulence factors, pathogenic potential, and host-specific traits associated with symptomatic infections are still not well understood. The growing number of immunocompromised patients has contributed to an increasing prevalence of *Blastocystis* spp. infections, which may be implicated in the development of various inflammatory diseases, including irritable bowel syndrome (IBS), colorectal cancer, and autoimmune disorders such as Hashimoto’s disease and ulcerative colitis. However, the presence of nonspecific symptoms often complicates diagnosis. This study aimed to present current data on the impact of *Blastocystis* spp. on the development and progression of gastrointestinal and autoimmune diseases, as well as to explore potential treatment options for *Blastocystis* spp. infections. A literature review was conducted to analyze the role of *Blastocystis* spp. in the pathogenesis of specific diseases and to investigate potential mechanisms of its interaction with the host organism. Advances in diagnostic techniques, particularly PCR, allow not only for the detection of *Blastocystis* spp. but also for the identification of specific subtypes, improving treatment precision. Beyond conventional therapies like metronidazole, there is a growing emphasis on alternative treatments, including the use of medicinal plants and probiotics.

## 1. Introduction

Protozoa of the genus *Blastocystis* are unicellular, anaerobic, eukaryotic organisms that colonize the gastrointestinal tract of various mammals, including humans. Although they were initially considered commensals of the large intestine due to their asymptomatic presence, subsequent clinical observations and patient-reported symptoms have suggested their potential pathogenicity. Today, *Blastocystis* spp. is recognized as a possible etiological agent of chronic diarrhea, particularly in immunocompromised individuals, as well as in patients with functional bowel disorders, malnutrition, cancer, or those who have undergone organ transplantation [1,2,3,4].

The current taxonomic classification of *Blastocystis* is as follows: Kingdom *Sar*, Phylum *Stramenopiles*, Class *Bigyra*, Order *Opalinata*, Family *Blastocystidae*, and Genus *Blastocystis* [5,6]. The species-level classification remains unresolved. Historically, species names were assigned based on the host from which the protozoan was isolated (e.g., *Blastocystis hominis*, *B. ratti*). Studies have shown that host specificity and pathogenic potential correlate with variations in the sequence of the small subunit ribosomal RNA (SSU rRNA) [1,7,8]. Molecular analysis of the SSU rRNA gene has identified at least 42 subtypes (STs) of *Blastocystis* spp. across various animals and humans [9], with some subtypes suspected to be pathogenic [10]. In humans, 16 subtypes have been identified to date, with ST1–ST4 being the most commonly associated with infection [9,11]. Given the unresolved species classification, the appropriate nomenclature is *Blastocystis* spp., with subtypes determined based on molecular SSU rRNA or SSU rDNA analysis [1].

*Blastocystis* spp. exhibit diverse morphological forms that vary in structure and size and can transition between forms in response to environmental factors. These include vacuolar, avacuolar, multivacuolar, granular, amoeboid, and cystic forms [12]. Transmission occurs via the fecal–oral route, with the cyst serving as the infectious stage. After ingestion, the cyst undergoes excystation in the host’s gastrointestinal tract, releasing vacuolar forms capable of binary fission. These forms subsequently encyst in the intestinal lumen, producing new cysts that are excreted in feces, completing the transmission cycle [1].

Human infection with *Blastocystis* spp. primarily results from ingestion of cysts present in contaminated water and food (e.g., improperly washed vegetables and fruits). Direct transmission through contact with animal reservoirs, including livestock (pigs, goats, sheep, cattle) and birds, is also possible [13,14]. The prevalence of infection is influenced by several risk factors, such as sanitation infrastructure, hygiene practices, age, overall health, nutritional status, and lifestyle habits, including hand hygiene and water consumption practices [15].

An estimated one billion people worldwide are infected with *Blastocystis* spp., with prevalence rates ranging from 1.5–10% in developed countries to 30–50% in developing regions [16]. In immunocompetent individuals, infections are often asymptomatic or manifest as mild, nonspecific symptoms such as bloating and intestinal cramps [17]. However, immunocompromised individuals—including those with HIV/AIDS, cancer, organ transplants, and those undergoing immunosuppressive therapy or hemodialysis—are particularly vulnerable. The estimated prevalence in this population averages 10% across high- and middle-income countries [18,19,20]. In these patients, *Blastocystis* spp. infection can lead to severe diarrhea due to progressive immune dysfunction [21]. Animal models suggest that impaired immunity exacerbates disease severity, as evidenced by extensive intestinal involvement and increased production of proinflammatory cytokines and antibodies compared to immunocompetent individuals [22].

Symptomatic blastocystosis may present as chronic diarrhea, abdominal pain, nausea, vomiting, anorexia, weight loss, and general weakness. Additionally, non-gastrointestinal symptoms such as rash, pruritus, and joint pain have been reported [3,23]. In patients with comorbidities or immunosuppression, *Blastocystis* spp. infections can be life-threatening [24].

Diagnosis primarily relies on direct microscopic examination of stool smears [23,25]. The vacuolar form is most frequently detected, whereas other morphological forms are more challenging to identify and may be confused with microorganisms, leukocytes, lipid droplets, or other fecal components [2]. Serological methods have limited utility, as their diagnostic value is still under investigation. Although culture methods offer high sensitivity, they are time-consuming and require specific growth conditions that are difficult to maintain [2,23]. Given these limitations, molecular diagnostic techniques, particularly polymerase chain reaction (PCR), are increasingly favored. PCR not only offers high sensitivity but also enables subtype differentiation [23,26]. Studies indicate that PCR is superior to traditional diagnostic methods for confirming *Blastocystis* spp. infections [27]. Furthermore, PCR techniques exhibit higher sensitivity, specificity, and predictive values compared to culture and microscopy [28,29].

The role of *Blastocystis* spp. in human gastrointestinal health remains a subject of ongoing scientific investigation [2]. Research is exploring its potential involvement in the pathogenesis of colorectal cancer, irritable bowel syndrome (IBS), and autoimmune diseases such as ulcerative colitis and Hashimoto’s thyroiditis [30,31,32,33].

## 2. Materials and Methods

This review was based on scientific publications retrieved from online databases, including PubMed, MDPI, NCBI, and Google Scholar, with the final search conducted in January 2025. Articles were identified using the following keywords: “*Blastocystis*”, “gastrointestinal diseases”, “irritable bowel syndrome”, “colorectal cancer”, “autoimmune diseases”, “treatment”, “metronidazole”, “medicinal plants”, and “probiotics”. This review encompasses comprehensive reviews, original research case reports, and articles written in English and published in peer-reviewed journals. We excluded brief communications and gray literature (e.g., conference proceedings and abstracts). Upon application of these criteria, a total of 154 papers were shortlisted for review. A narrative review was then conducted, selecting the most relevant and informative studies for inclusion.

This study aimed to present current data on the impact of *Blastocystis* spp. on the development and progression of gastrointestinal and autoimmune diseases, as well as to explore potential treatment options for *Blastocystis* spp. infections. A literature review was conducted to analyze the role of *Blastocystis* spp. in the pathogenesis of specific diseases and to investigate potential mechanisms of its interaction with the host organism.

## 3. *Blastocystis* spp. and Gastrointestinal Diseases

### 3.1. Irritable Bowel Syndrome (IBS)

Irritable bowel syndrome is a chronic functional gastrointestinal disorder affecting 10–20% of the population, with the highest prevalence reported in Western Europe and North America [29,34]. IBS is characterized by altered bowel movement patterns, changes in stool consistency, bloating, excessive gas production, and abdominal pain associated with either diarrhea or constipation [29,35,36]. The exact etiology of IBS remains unclear, but it is believed to involve a combination of psychosocial factors, altered gut motility and hypersensitivity, and disturbances in the gut microbiota [29]. Studies suggest that IBS can develop following gastrointestinal infections (post-infectious IBS) or as a consequence of prolonged broad-spectrum antibiotic use [36,37,38]. Environmental and dietary factors have also been implicated in IBS pathogenesis [35].

Recent research has established a link between parasitic infections and IBS symptoms. A study by Das et al. [39] found that 56% of IBS patients were co-infected with gastrointestinal parasites, including *Giardia intestinalis* and *Entamoeba histolytica*. The high prevalence of *Blastocystis* spp. in IBS patients further suggests a potential role in the disorder’s pathogenesis [39,40,41,42]. For instance, Ibrahim et al. [43] reported a significantly higher prevalence of *Blastocystis* spp. in IBS patients (33.5%) compared to the control group (12%). Nahhas [44] observed an even higher infection rate, with *Blastocystis* spp. detected in 71.4% of IBS patients. Additionally, a reduction in IBS symptoms following antiparasitic treatment further supports the possible involvement of *Blastocystis* spp. in IBS pathogenesis. Kesuma et al. [31] demonstrated an association between *Blastocystis* ST1 and diarrhea-predominant IBS in Indonesian adolescents, where *Blastocystis* spp. was identified in 36.5% of IBS patients, and diarrhea occurred three times more frequently in individuals infected with *Blastocystis* ST1.

Despite these findings, it remains unclear whether IBS-related gut dysfunction facilitates *Blastocystis* spp. colonization or whether the presence of the protozoan contributes to intestinal disturbances, ultimately leading to IBS symptoms [36].

Intestinal parasites may influence gastrointestinal function by increasing gut permeability, triggering immune responses, and promoting chronic inflammation [45,46]. Epithelial barrier dysfunction has been observed in duodenal tissue from patients with chronic giardiasis [47], while studies in animal models have demonstrated altered intestinal motility and visceral sensitivity in *Trichinella spiralis* infection [48]. Several mechanisms have been proposed to explain *Blastocystis* spp. interactions with the immune system and their effects on gut function. One such mechanism involves the ability of *Blastocystis* spp. to produce cysteine proteases that degrade glycoproteins within mucin, a key component of the gastrointestinal mucus layer [35]. Mucin plays an essential role in maintaining gut hydration, protecting epithelial cells from stress, and preventing pathogen infection [49]. Degradation of mucin may initiate inflammatory and allergic responses [35]. Moreover, *Blastocystis* spp. has been shown to disrupt the integrity of the intestinal barrier by modulating tight junction proteins (TJs), such as claudin-7, leading to increased epithelial permeability. This disruption may heighten intestinal sensitivity to external stimuli, potentially contributing to IBS symptoms [36].

A correlation has also been identified between *Blastocystis* spp. infection and elevated proinflammatory cytokine levels. Ragavan et al. [29] reported significantly higher levels of interleukins (IL-3, IL-5, and IL-8) in IBS patients infected with *Blastocystis* spp. compared to uninfected individuals. Similarly, Ismail et al. [50] found increased plasma concentrations of IL-6, IL-8, IL-10, IFN-γ, and TNF-α in IBS patients with *Blastocystis* spp. infection. Furthermore, single nucleotide polymorphisms (SNPs) in IL-8 and IL-10 have been suggested as potential risk factors for IBS development in infected individuals [51]. Hussain et al. [52] provided further support for this hypothesis, observing elevated antibody levels against *Blastocystis* spp. in IBS patients compared to control subjects.

Dysbiosis has also been proposed as a contributing factor to IBS [53] and *Blastocystis* spp. may influence both the composition and diversity of gut microbiota. Studies have shown that *Blastocystis* spp. can reduce the abundance of beneficial bacteria such as *Lactobacillus* spp. and *Bifidobacterium* spp. [54]. Additionally, its presence has been associated with a decreased *Firmicutes*/*Bacteroidetes* ratio in individuals with metabolic disorders compared to healthy controls [55]. Some studies have also suggested a synergistic relationship between *Blastocystis* spp. and *Clostridium difficile*, with both organisms being co-detected in patients with diarrhea [56,57].

Although many studies support an association between IBS and *Blastocystis* spp., others have failed to establish a significant correlation [58,59]. Further large-scale studies are needed to elucidate the precise role of *Blastocystis* spp. in IBS pathogenesis.

### 3.2. Blastocystis spp. and Colorectal Cancer (CRC)

Colorectal cancer (CRC) is the third most frequently diagnosed malignancy and the second leading cause of cancer-related mortality worldwide [60]. Its development is influenced by a range of risk factors, including chronic infections and inflammation, poor dietary habits, stress, and prolonged exposure to radiation and toxic chemicals [61]. Infectious agents, including parasites, are estimated to contribute to approximately 16% of all cancers [62]. Several bacterial species, such as *Fusobacterium nucleatum*, *Bacteroides fragilis*, *Escherichia coli*, and *Helicobacter pylori*, have been implicated in the initiation and progression of CRC [63]. Similarly, the increased prevalence of *Blastocystis* spp. in CRC patients suggests a potential role of this protozoan in carcinogenesis [64].

A review of the literature indicates that *Blastocystis* spp. is detected more frequently in CRC patients than in healthy individuals, with reported prevalence rates ranging from 2.8% to 52%, predominantly involving subtypes ST1 and ST3 [30,65,66]. Notably, Ali et al. [65] were the first to report the presence of the less common subtype ST7 in three patients with colonic adenocarcinoma (grade 2) and a history of colectomy. This subtype has been associated with gut microbiota dysbiosis and a reduction in beneficial bacterial populations, such as *Lactobacillus* spp. and *Bifidobacterium* spp. [54,67]. In CRC patients, *Blastocystis* spp. has been identified in colonic washouts (12.47%) and fecal samples (6.12%) [68]. Interestingly, chemotherapy does not appear to influence the presence of *Blastocystis* spp. [64]. Additionally, *Blastocystis* spp. has been documented in advanced CRC stages (grades 3 and 4), where its presence correlates with elevated levels of proinflammatory cells and increased plasma TNF-α [69]. These findings suggest that tumor-induced changes in the colonic environment may facilitate *Blastocystis* spp. colonization.

Conversely, several researchers propose that *Blastocystis* spp. is not merely an opportunistic colonizer but may act as a contributing factor to CRC development. Kumarasamy et al. [70] conducted an animal model study comparing the effects of azoxymethane (AOM), a known carcinogen, with the combined exposure to AOM and *Blastocystis* spp. cysts (ST3). Their findings revealed that simultaneous exposure to ST3 and AOM led to a twofold increase in the number of aberrant crypt foci in the colon compared to rats exposed to AOM alone. It has also been suggested that *Blastocystis* spp. may induce oxidative stress, disrupt epithelial homeostasis, and promote intestinal barrier dysfunction, all of which contribute to CRC pathogenesis [64].

In vitro studies have further demonstrated that *Blastocystis* spp. enhances the proliferation of HCT116 colorectal cancer cells. This effect may be mediated through the activation of immune regulatory proteins, such as cathepsin B, which has been identified as a key factor in CRC progression, invasion, and metastasis [71,72]. Moreover, *Blastocystis* spp. infections have been linked to the suppression of nitric oxide synthase (NOS), leading to a reduction in nitric oxide levels, which are essential for immune defense and tumor suppression [73].

**Figure 1:**
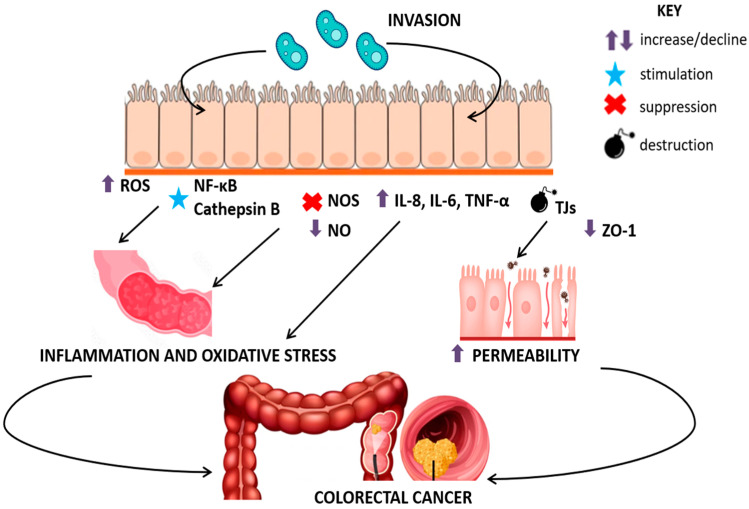
Possible mechanisms of *Blastocystis* spp.-mediated colorectal cancer (own elaboration) (ROS, reactive oxygen species; NF-κB, nuclear factor kappa B; NOS, nitric oxide synthase; NO, nitric oxide; IL-8, interleukin-8; IL-6, interleukin-6; TNF-α, tumor necrosis factor alpha; TJs, tight junctions; ZO-1, zonula occludens-1 protein.

Another mechanism by which *Blastocystis* spp. may contribute to CRC is its ability to trigger the release of proinflammatory cytokines, including IL-8, IL-6, IL-1β, and TNF-α, which activate multiple signaling pathways involved in tumorigenesis (Figure 1). This process may lead to increased stem cell activity, enhanced cellular proliferation and migration, and the promotion of angiogenesis, all of which support cancer progression [64].

Additionally, *Blastocystis* spp. has been shown to compromise intestinal barrier integrity by disrupting tight junction (TJ) proteins, such as claudins and occludins, which maintain epithelial cohesion [74]. Increased epithelial permeability can contribute to leaky gut syndrome, a recognized risk factor for CRC [75]. Furthermore, *Blastocystis* spp. has been implicated in the downregulation of zonula occludens-1 (ZO-1), a tumor suppressor protein. Reduced ZO-1 expression results in a weakened epithelial barrier and increased colorectal cancer cell proliferation [64]. Similarly, *Blastocystis hominis* and *Blastocystis ratti WR1* have been shown to stimulate the release of inflammatory cytokines, particularly IL-8, via NF-κB activation [76]. Evidence also suggests that *Blastocystis* spp. may promote colorectal cancer cell proliferation by dysregulating IFN-γ and p53 expression, further highlighting its potential role in carcinogenesis [77].

Despite the strong association between *Blastocystis* spp. infections and CRC observed in various studies, many questions remain unanswered. The specific role of the protozoan in different stages of CRC progression has yet to be fully elucidated, and it is unclear whether *Blastocystis* spp. alone can initiate malignant transformation [30]. Current data remain insufficient to draw definitive conclusions regarding the significance of *Blastocystis* spp. in CRC, emphasizing the need for further research.

## 4. *Blastocystis* spp. in Autoimmune Diseases

Autoimmune diseases result from a complex interplay of genetic and environmental factors, leading to excessive immune activation and the production of autoantibodies against self-tissues [78,79]. Similarly, many pathogenic and virulent factors can trigger autoimmune reactions through various mechanisms [80]. Changes in the gut microbiota composition, including those induced by *Blastocystis* spp., have been identified as potential contributors to immune dysregulation and the onset of autoimmune diseases [67,81].

The involvement of *Blastocystis* spp. in autoimmune thyroiditis, particularly Hashimoto’s disease, has been highlighted in several studies [33,82]. Interleukin-17 (IL-17) is considered a key factor in the disease’s pathogenesis, alongside elevated levels of anti-thyroid peroxidase (anti-TPO) antibodies and increased thyroid-stimulating hormone (TSH), which indicate thyroid dysfunction [33,83,84]. El-Zawawy et al. [33] observed significantly higher IL-17 levels in the plasma of Hashimoto’s patients infected with *Blastocystis* spp. compared to uninfected individuals. Notably, *Blastocystis* spp. eradication led to a decrease in IL-17 levels and an improvement in thyroid function parameters. Similar findings were reported by Rajič et al. [82], who described a 49-year-old male with Hashimoto’s disease, where *Blastocystis* spp. eradication resulted in normalized thyroid hormone levels, reduced anti-thyroid antibody concentrations, and the resolution of disease symptoms.

There is also evidence linking *Blastocystis* spp. to cutaneous lesions and urticaria [85]. Chronic spontaneous urticaria (CSU) is a condition characterized by recurrent wheals and/or angioedema persisting for at least six weeks. The underlying causes are believed to include autoimmune reactions, food intolerances, bacterial and viral infections, and parasitic infestations [86]. Jafari et al. [87] found a significantly higher prevalence of *Blastocystis* spp. in urticaria patients compared to controls. While both groups harbored ST1, ST2, and ST3, no correlation was identified between specific subtypes and symptom severity. However, Aykur et al. [88] observed a strong association between ST3 and CSU development. Additionally, amoeboid forms of *Blastocystis* spp., which are considered the most virulent, were predominantly isolated from patients with chronic urticaria [89,90]. Amoeboid *Blastocystis* forms are thought to adhere more effectively to intestinal epithelial cells, disrupt gut homeostasis, and trigger immune responses against parasite surface antigens, leading to inflammatory cell recruitment [90]. This immune activation promotes histamine release, which may contribute to allergic reactions and chronic urticaria [91].

The presence of *Blastocystis* spp. has also been documented in patients with ulcerative colitis (UC), a chronic inflammatory bowel disease (IBD) of unknown etiology, characterized by mucosal and submucosal inflammation of the colon [92]. However, multiple studies have found no clear association between *Blastocystis* spp. and UC development [32,93]. Kök et al. [32] reported no significant difference in *Blastocystis* spp. prevalence between remission and active disease phases. Interestingly, UC patients infected with *Blastocystis* spp. exhibited milder symptoms, suggesting a potential protective effect [32]. In contrast, Rossen et al. [94] observed a lower prevalence of *Blastocystis* spp. in UC patients compared to healthy individuals, possibly due to increased protozoan clearance in response to chronic intestinal inflammation [94].

The role of *Blastocystis* spp. in autoimmune diseases remains uncertain. While its immune-modulating effects may contribute to Hashimoto’s disease and chronic urticaria, evidence suggests a potential protective role in ulcerative colitis. Due to the limited data available, further research is necessary to clarify the significance of *Blastocystis* spp. in autoimmune disease pathogenesis.

The prevalence of *Blastocystis* spp. in various gastrointestinal and autoimmune diseases, along with the most frequently detected subtypes, is summarized in Table 1. Differences in reported prevalence may stem from disease-specific factors, sample size variations, and discrepancies in diagnostic methodologies. The most commonly employed diagnostic methods for *Blastocystis* spp. infections in patients with gastrointestinal and autoimmune diseases include microscopic examination using various staining techniques and molecular methods, particularly polymerase chain reaction (PCR). Keshawy and Alabbassy [95] demonstrated that PCR-based detection was more accurate than direct microscopy, identifying *Blastocystis* spp. in 10 out of 24 samples, whereas microscopy detected only 2 positive cases. Additionally, PCR enables subtype differentiation, which has revealed that ST1, ST2, and ST3 are the most prevalent subtypes in gastrointestinal and autoimmune diseases, though minor variations in subtype distribution exist across studies [87,96,97].

*Blastocystis* spp. infections often present with nonspecific gastrointestinal symptoms, including diarrhea, irregular bowel movements, bloating, and abdominal pain. However, in patients with chronic urticaria, gastrointestinal symptoms are rarely observed.

**Table 1:** Prevalence of *Blastocystis* spp. in gastrointestinal and autoimmune diseases (SLE, systemic lupus erythematosus; IBS, irritable bowel syndrome; UC, ulcerative colitis; CD, celiac disease; CRC, colorectal cancer; DM, direct microscopy; PCR, polymerase chain reaction).

Condition	Symptoms	Samples (n)	Positive *Blastocystis* Samples (n)	Prevalence (%)	Diagnostic Method (Stool Examination)	Most Common Subtype	References
SLE	Inflammation, vasculitis, immune complex deposition, vasculopathy	38	5	13.2	DM (trichrome staining), PCR	No data	[98]
SLE and IBS	No data	24	10	41.6	PCR	ST3	[95]
			2	8.3	DM (saline/iodine)		
UC	No data	21	5	23.8	DM (trichrome staining), PCR	No data	[98]
UC	Acute diarrhea, loss of appetite, dyspepsia, constipation, abdominal pain	276	24	8.7	Native-Lugol, formol = ethyl acetate concentration	No data	[99]
UC	No data	150	12	8	DM, Jones’ medium culture, PCR	ST3	[100]
IBS	Abdominal pain or discomfort, improvement with defecation, change in frequency of defecation, change in stool form	122	24	19.7	DM (trichrome staining), PCR	ST3	[59]
IBS	Recurrent abdominal pain, improvement with defecation, change in frequency of defecation, change in stool form	115	22	19.1	DM (wet mount, trichrome staining), Jones’ medium culture, PCR	ST3	[101]
IBS	Diarrhea, constipation, abdominal pain, flatulence, weight loss, nausea, vomiting	35	25	71.4	DM, PCR	ST1	[44]
CD	No data	92	15	16.3	PCR	ST1, ST2, ST3	[97]
CD	No data	75	31	41.3	DM, PCR	ST3	[102]
CRC	No data	15	9	60	DM (Wheatley Trichrome), PCR	ST2	[103]
CRC	No data	74	22	29.7	Jones’ medium culture, DM, PCR	ST1	[66]
Urticaria	Skin lesions with or without gastrointestinal symptoms	54	33	61.1	DM, PCR	ST3	[89]
Urticaria	Skin lesions	135	43	31.9	Direct saline smear, Lugol’s iodine staining, trichrome staining, Jones’ medium culture, PCR	ST3	[88]
Urticaria	Skin lesions	94	20	21.3	DM, PCR	ST1, ST2, ST3	[87]
Spondyloarthritis	Diarrhea, mucus in stool, hematochezia, increased frequency of daily bowel movements, abdominal pain, abdominal distension	74	50	67.6	PCR	ST1, ST2, ST3	[96]

## 5. Treatment of *Blastocystis* spp. Infections

### 5.1. Conventional Treatment

In many cases, *Blastocystis* spp. infections do not require treatment due to their self-limiting nature. However, therapy is recommended for patients experiencing persistent and severe symptoms that significantly impair daily functioning [18,104,105]. Various antimicrobial agents have been used to treat *Blastocystis* spp. infections, with varying degrees of effectiveness. Currently, metronidazole, a nitroimidazole derivative, is considered the drug of choice. Its mechanism of action involves disrupting microbial DNA and inducing cell death [104,105].

Metronidazole is typically administered at doses ranging from 250 to 750 mg three times daily or 1500 mg once daily for ten days. Combination therapies, such as metronidazole with trimethoprim–sulfamethoxazole or paromomycin, have also been increasingly used [18]. Several studies have highlighted the high efficacy of metronidazole and its superiority over other antimicrobial agents [106,107,108,109,110]. Following metronidazole therapy, approximately 90% of patients achieve disease remission with no recurrence within six months [105]. However, literature reviews indicate that the eradication rate of *Blastocystis* spp. varies widely, ranging from 0% to 100% [104,105,111].

**Figure 2:**
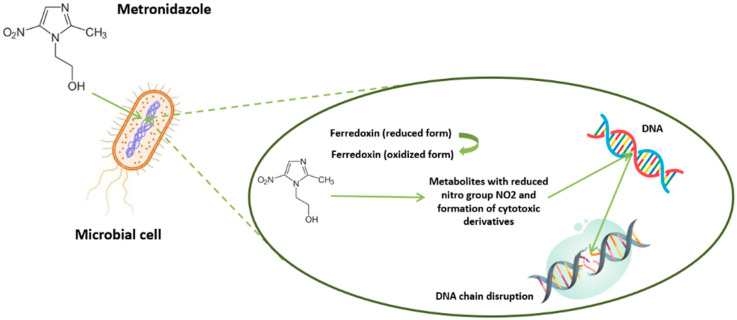
Mechanism of metronidazole action (own elaboration).

The variability in treatment response may be due to regional differences in drug susceptibility, as well as emerging resistance to chemotherapeutic agents [18,104,105,111,112]. Certain *Blastocystis* spp. subtypes may exhibit natural resistance to metronidazole, or their susceptibility may depend on drug concentration [104,113,114]. The cystic form of *Blastocystis* spp. may also be resistant to metronidazole’s cytotoxic effects, as its thick-walled structure allows survival under extreme environmental conditions [104,105]. Studies have shown that cysts can withstand metronidazole concentrations of up to 5 mg/mL [113]. Another possible explanation for metronidazole resistance in *Blastocystis* spp. is reduced activity of the pyruvate:ferredoxin oxidoreductase enzyme, which is required for activating the drug (Figure 2) [115]. This mechanism has also been observed in other protozoan parasites, including metronidazole-resistant *Giardia intestinalis* [116].

The occurrence of adverse effects is another limitation of metronidazole therapy. The drug may cause gastrointestinal disturbances, and in rare cases, long-term or high-dose use can lead to serious health complications. Severe side effects include neurotoxicity, hepatic encephalopathy, peripheral neuropathy, and optic neuropathy [117]. There is also evidence suggesting that metronidazole possesses carcinogenic, teratogenic, embryotoxic, and genotoxic potential [117,118].

Recent research suggests that metronidazole resistance may increase the pathogenicity of *Blastocystis* spp. Rajamanikam et al. [119] examined parasite growth, apoptosis, protease activity, and the ability of *Blastocystis* spp. to promote cancer cell proliferation after exposure to metronidazole at a concentration of 0.001 mg/mL. Their findings showed an increase in the number of parasites, particularly amoeboid forms, as well as a significant rise in cysteine protease activity, an enzyme playing a crucial role in the *Blastocystis* spp. cell cycle. Additionally, an enhanced ability to promote cancer cell proliferation was observed, indicating a higher pathogenic potential in resistant strains. In contrast, Wu et al. [74] found that metronidazole-resistant ST7 strains exhibited reduced pathogenicity, likely due to impaired adhesion to intestinal epithelial cells.

The successful elimination of *Blastocystis* spp. depends on maintaining a sufficient drug concentration in the intestine to effectively destroy the protozoan [104]. In addition to metronidazole, trimethoprim–sulfamethoxazole and nitazoxanide have demonstrated similar efficacy and are currently considered second-line treatments for blastocystosis. Both drugs are well tolerated and do not typically cause severe adverse effects [105]. Paromomycin, a broad-spectrum aminoglycoside antibiotic, has also been used to treat skin lesions associated with *Blastocystis* spp. infections [120]. Several other drugs, including tinidazole, ketoconazole, pentamidine, quinine, iodoquinol, furazolidone, and emetine, have shown varying degrees of effectiveness and may be considered alternative options when metronidazole and second-line therapies fail [121].

Despite the availability of multiple chemotherapeutic agents active against *Blastocystis* spp., complete eradication remains challenging. The primary obstacles to treatment success include variability in drug susceptibility among different *Blastocystis* subtypes, geographic differences in resistance patterns, and the emergence of drug-resistant strains. Additionally, factors such as poor adherence to treatment regimens, differences in drug pharmacokinetics, and inactivation of therapeutic compounds by the host’s natural microbiota further contribute to treatment failure [105]. Given these limitations, researchers have increasingly turned their attention to alternative therapeutic strategies, particularly natural compounds with potential antiparasitic properties.

### 5.2. Alternative Treatments

Recent studies have explored the potential of natural compounds and medicinal plants as alternative therapies for *Blastocystis* spp. infections. Certain plant extracts and dietary components have demonstrated antiparasitic activity, which may aid in the eradication of the protozoan (Table 2).

One of the most promising medicinal plants is *Salvadora persica* L., widely used as a natural oral hygiene agent. Studies have shown that aqueous extracts from its roots exhibit strong activity against *Blastocystis* subtypes ST1, ST3, and ST5. Inhibition of *Blastocystis* spp. growth reached 80% after 48 h of incubation in an extract concentration of 40 µL/mL. Notably, *S. persica* extracts retained their protozoicidal properties even at high temperatures [122].

Traditional Chinese medicine has also provided insights into potential anti-*Blastocystis* treatments. Extracts from linalool (*Boesenbergia rotunda* (L.) Mansf.) and *Ganoderma lucidum* (Fr.) Kart have demonstrated amoebicidal activity. When combined with metronidazole at a concentration of 62.5 µg/mL, these extracts inhibited protozoan growth by up to 90% within 12 h [123]. Their active components, including geraniol, camphor, linalool, and versalide (*Ganoderma lucidum*), are thought to be responsible for the observed antiparasitic effects.

Another promising plant is *Eurycoma longifolia*, known as Tongkat Ali. Alcoholic extracts of this plant have exhibited activity against *Blastocystis* spp. comparable to metronidazole [124]. The primary bioactive compound, eurycomanone, is believed to be mainly responsible for its protozoicidal effects. Unlike metronidazole, Tongkat Ali extracts appear to be effective against multiple *Blastocystis* spp. subtypes without requiring subtype identification, which could significantly streamline the diagnostic process [125].

Medicinal plants from Egypt have also been investigated for their anti-*Blastocystis* potential. *Achillea fragrantissima* (Forssk.) Sch. Bip. has demonstrated activity against ST1 and ST3 subtypes. At a concentration of 4000 µg/mL, its extracts induced morphological changes in *Blastocystis* spp., leading to complete protozoan destruction after 72 h [126]. Similarly, *Origanum majorana* L. and *Foeniculum vulgare* Mill. have shown antioxidant and antiparasitic properties, with *O. majorana* extract at 400 µg/mL exhibiting protozoicidal effects comparable to nitazoxanide at 500 µg/mL [127].

Additionally, several medicinal plants from Ghana have been reported to exhibit strong activity against *Blastocystis* spp., including *Mallotus oppositifolius* (Geiseler) Müll. Arg., which has shown efficacy comparable to metronidazole [128]. Ahmed et al. [129] further expanded the list of plants with cytotoxic effects on *Blastocystis* spp. by identifying *Ptilostemon chamaepeuce* subsp. *cyprius*, a species endemic to Cyprus, as a potential therapeutic candidate.

Dietary ingredients and spices have also been studied for their potential in *Blastocystis* spp. eradication. Extracts from garlic (*Allium sativum* L.), ginger (*Zingiber officinale* Rosc.), horseradish (*Armoracia rusticana* B. Mey et Scherb.), and turmeric (*Curcuma longa* L.) have shown varying degrees of efficacy against ST3 and ST7. Garlic and turmeric were the most effective against ST3, while turmeric and horseradish showed the greatest reduction in ST7 populations [130]. Garlic extracts, rich in thiosulfonates such as allicin, inhibit protein and nucleic acid synthesis in *Blastocystis* spp., significantly reducing parasite counts after 48 h of incubation, with effects comparable to metronidazole and nitazoxanide [131,132].

Hexahydrocurcumin, a bioactive compound found in ginger extracts, has been shown to possess protozoicidal properties [117]. Abdel-Hafeez et al. [133] reported that treatment with garlic and ginger extracts significantly reduced the number of *Blastocystis* spp. cysts excreted by mice compared to the control group. Both garlic and ginger are potent antioxidants that can inhibit the production of nitric oxide (NO), a compound whose persistently elevated levels during *Blastocystis* spp. infection contribute to intestinal barrier damage [117,133]. In contrast, black pepper (*Piper nigrum* L.) and white cumin (*Trachyspermum ammi* L.) are considered to have a more limited effect on *Blastocystis* spp., and further research is needed to assess their potential efficacy [132].

Drugs commonly used for other medical conditions may also aid in the eradication of protozoan infections. Basyoni et al. [134] investigated the efficacy of atorvastatin, a statin drug, in combating *Blastocystis* spp. infections. In addition to their cholesterol-lowering effects, statins have been suggested to protect intestinal barrier integrity by inhibiting specific enzymes [135]. Atorvastatin, administered at doses of 20–40 mg/kg, exhibited strong activity against *Blastocystis* spp., and when combined with metronidazole (10 mg/kg), it achieved a 98–99% reduction in parasite cell numbers [134].

Simeprevir, a serine protease inhibitor primarily used in the treatment of hepatitis C virus (HCV) infections, has also been studied for its potential anti-*Blastocystis* effects [136]. Serine proteases, which play a critical role in HCV maturation, are also involved in the regulation of proinflammatory cytokines during *Blastocystis* spp. infections [136,137]. Increasing concentrations of simeprevir were found to progressively inhibit the growth and viability of *Blastocystis* spp. The drug’s mechanism of action was attributed to the induction of cell membrane rupture, ultimately leading to necrotic cell death [136].

### 5.3. The Role of Probiotics in Therapy

Probiotics have been shown to reduce the number of parasites and alleviate symptoms associated with their colonization. Clinical studies support their use as adjunctive therapeutic agents in parasitic infections [138,139]. Probiotics are live microorganisms that, when consumed as part of the diet, can influence the composition of the gut microbiota [34]. Since *Blastocystis* spp. infections have been linked to gut microbiota imbalances, probiotic therapy may contribute to restoring microbial homeostasis [6].

Among the most studied probiotics for *Blastocystis* spp. infections is *Saccharomyces boulardii*, which has demonstrated effectiveness in treating gastrointestinal disorders with an inflammatory component [105,139]. Clinical research has confirmed its ability to alleviate symptoms such as diarrhea, vomiting, abdominal pain, and bloating in children infected with *Blastocystis* spp. [140]. Similar improvements were observed by Angelici et al. [141], who reported complete resolution of gastrointestinal symptoms in a patient following *S. boulardii* supplementation. Animal studies have further shown that live *S. boulardii* strains significantly reduce levels of proinflammatory cytokines (IL-6, IL-8, TNF-α) and inhibit the expression of inducible nitric oxide synthase (iNOS) in the colonic mucosa of rats infected with *Blastocystis* spp. [142]. Additionally, certain bacterial strains, including *Lactobacillus rhamnosus*, *Lactococcus lactis*, and *Enterococcus faecium*, have been found to inhibit the growth of *Blastocystis* ST3 [143].

**Table 2:** Natural plant extracts and probiotics as alternative treatment options against *Blastocystis* sp. infection.

Name	Part Used	*Blastocystis*			Reference
		Source	Subtype	Form Susceptible to the Treatments	
***Plants***					
*Salvadora persica* L.	root	clinical isolate (gastrointestinal complaints)	ST1, ST3, ST5		[122]
*Boesenbergia rotunda* (L.) Mansf	rhizome	clinical isolate	ST3	vacuolar forms	[123]
*Ganoderma lucidum* (Fr.)	fruiting body		ST3		
*Eurycoma longifolia* (Tongkat Ali)	root	clinical isolate	ST1, ST3, ST5		[124]
*Achillea fragrantissima* (Forssk.) Sch. Bip. (Qaysoom)	aerial parts	clinical isolate (gastrointestinal symptoms)	ST1, ST2	vacuolar/granular forms	[126]
*Origanum majorana* L. (Marjoram)	leaves	clinical isolate (diarrhea samples)		cyst	[127]
*Foeniculum vulgare* Mill.	seeds				
*Mallotus oppositifolius* (Geiseler) Müll	cortex and radix	clinical isolate	ST4		[128]
*Ptilostemon chamaepeuce* subsp. *cyprius*	leaves	clinical isolate (abdominal pain)	ST1, ST1 and ST3 coinfection	vacuolar/granular forms	[129]
*Allium sativum* L. (Garlic)	cloves	clinical isolate (symptomatic individuals: ST3-intestinal methanogen overgrowth (IMO), ST7-rectal cancer)	ST1 (x), ST7	vacuolar forms	[130]
*Zingiber officinale* Rosc. (Ginger)	roots		ST1, ST7 (x)		
*Armoracia rusticana Gaertn*. (Horseradish)	roots		ST3		
*Curcuma longa* L. (Turmeric)	turmeric powder		ST3 (x), ST7		
*Allium sativum* L. (Garlic)	fresh bulbs of garlic	clinical isolate (patients with irritable bowel syndrome (IBS))	ST1, ST1 and ST3 coinfection	vacuolar forms	[132]
*Allium sativum* L. (Garlic)	fresh peeled cloves	mice infected with *Blastocystis* (experimental model)		cyst	[133]
*Zingiber officinale* Rosc. (Ginger)	ginger powder				
**Probiotics**					
*Saccharomyces boulardii*		clinical isolate (gastrointestinal symptoms)		cyst (*S. boulardii* has potential beneficial effects)	[140]
*Lactobacillus rhamnosus*		culture	ST3	inhibition of *Blastocystis* proliferation by LAB	[143]
*Lactococcus lactis*			ST3		

x—the highest inhibitory effect.

The mechanisms through which probiotics exert their effects are complex. Different probiotic strains, including bacterial and yeast-based probiotics, influence the immune system by modulating host immune responses [139]. Their interaction with intestinal epithelial and immune cells enhances the production of immunoglobulins IgA and IgM, strengthening mucosal immunity and reinforcing the barrier against pathogenic microorganisms [144,145]. *S. boulardii* has been shown to regulate cytokine levels, increasing the ratio of anti-inflammatory cytokines (IL-4, IL-10) to proinflammatory cytokines (IL-8, IL-1β), thereby reducing inflammation [146].

Another key probiotic mechanism involves microbial competition for ecological niches within the gut. Probiotics such as *Lactobacillus* spp. can deprive pathogens of essential nutrients by binding and transporting iron compounds, making them unavailable for pathogen growth [139]. Some probiotic species also produce bioactive compounds, including bacteriocins, organic acids, and hydrogen peroxide, which inhibit the survival and replication of pathogens in the gastrointestinal tract [139,147,148]. *Lactobacillus reuteri* produces reuterin (3-hydroxypropionaldehyde), a broad-spectrum antimicrobial agent effective against bacteria, viruses, yeasts, fungi, and protozoa [149]. Additionally, lactic acid bacteria inhibit acid-sensitive microorganisms by producing lactic acid, which lowers the local pH and disrupts pathogen metabolism [150]. *S. boulardii* secretes antimicrobial peptides of varying molecular weights that reduce pathogen adherence to the intestinal epithelium and neutralize microbial toxins [146].

Probiotics also contribute to gut health by modulating the synthesis of short-chain fatty acids (SCFAs), including acetate, propionate, and butyrate. These metabolites play a crucial role in maintaining intestinal homeostasis and supporting key physiological and biochemical processes [146]. *S. boulardii* has been shown to restore normal SCFA levels, which are typically reduced during inflammatory conditions such as irritable bowel syndrome (IBS) [34,151,152].

The multifaceted effects of probiotics make them increasingly valuable as adjunctive treatments for various gastrointestinal disorders, with strong scientific evidence supporting their therapeutic efficacy. Their widespread availability in diverse formulations, including capsules, tablets, powders, drops, and pastes, allows for personalized treatment options based on patient age and preferences. Probiotics present a safe and promising alternative for managing pathogenic infections; however, excessive self-administration may lead to adverse effects, such as allergic reactions, sepsis, or endocarditis in individuals with severe gastrointestinal conditions. To minimize risks, it is essential to adhere to medical recommendations regarding dosage and duration of probiotic use. Further research is needed to refine our understanding of probiotic applications in human health and optimize their use in clinical practice [34,153,154].

## 6. Conclusions

The pathogenicity of *Blastocystis* spp. remains unresolved, and its virulence factors, pathogenic potential, and host-specific traits associated with symptomatic infections are still poorly understood.

1. This review highlights the often-overlooked association between *Blastocystis* spp. infections and gastrointestinal and autoimmune diseases. Patients with autoimmune disorders, where the immune system mistakenly attacks self-tissues, appear to be at a significantly higher risk of *Blastocystis* spp. infection compared to healthy individuals. Additionally, the increased prevalence of *Blastocystis* spp. in colorectal cancer (CRC) patients suggests a potential role in carcinogenesis, although further studies are necessary to confirm this hypothesis.
2. Symptomatic blastocystosis can manifest as chronic diarrhea, abdominal pain, nausea, vomiting, anorexia, weight loss, and general weakness. However, *Blastocystis* spp. infections are often characterized by nonspecific gastrointestinal symptoms, including diarrhea, irregular bowel movements, bloating, and abdominal discomfort. In contrast, patients with chronic urticaria rarely exhibit gastrointestinal symptoms.
3. The diagnosis of *Blastocystis* spp. infections still relies predominantly on microscopic methods, which are known for their low sensitivity. Therefore, a combination of direct microscopic examination and molecular techniques, particularly PCR, should be employed whenever possible to improve diagnostic accuracy.
4. Metronidazole remains the primary treatment for *Blastocystis* spp. infections, despite reports of treatment failure in some cases. When metronidazole proves ineffective, nitazoxanide may be considered as an alternative therapy.
5. Natural plant extracts and certain dietary components have demonstrated antiparasitic activity against *Blastocystis* spp. and may serve as adjunctive or alternative treatment options.

## Data Availability

Not applicable.
